# Influence of Ocular Surface Hydration on Intra-Operative Aberrometry Measurement and Toric Intraocular Lens Recommendation

**DOI:** 10.1167/tvst.11.11.18

**Published:** 2022-11-22

**Authors:** George He, Chandra Balachandran

**Affiliations:** 1 Royal Prince Alfred Hospital, Sydney, Australia; 2 Personal Eyes, Consultant for Alcon and Johnson & Johnson, Sydney, Australia

**Keywords:** intra-operative aberrometry, ocular surface, intraocular lens

## Abstract

**Purpose:**

The purpose of this study was to examine the effect of intra-operative live refraction stability as a surrogate marker of ocular surface hydration on intra-operative aberrometry (IA) results and to quantify the minimum duration of stable refraction needed to achieve accurate intraocular lens (IOL) selection.

**Methods:**

In this nonrandomized consecutive retrospective chart review, 18,000 data points from 45 live refraction runs of 15 patients were digitized and analyzed. An objective automated moving average method of frames lengths of 88 ms, 110 ms, and 132 ms with less than 0.5 diopters (D) of fluctuation was compared to raw IA capture data. The difference-vector (DV) of the predicted toric powers was compared among these groups. Subjectively, traces were classified as stable or unstable if the live refraction fluctuated less than 0.5 D for 5 seconds. The DV based on the stable period was compared with the IA capture data.

**Results:**

The DVs for all frame intervals showed no significant difference when compared with IA readings. In 15 of 45 (33.3%) cases, IA active refraction traces were stable and the DV (0.27 D ± 0.15 D) was significantly less than unstable traces (0.49 D ± 0.28 D). IOL recommendations from 14 (93.3%) of stable runs led to a <0.5 D of postoperative cylinder compared to 14 (47.7%) in unstable runs.

**Conclusions:**

Intra-operative live refraction stability is affected by ocular hydration. Surgeons should look for periods of refractive stability for at least 5 seconds to better assess measurement quality. This can be facilitated by capturing and including the active refraction trace, which is currently unavailable for review.

**Translational Relevance:**

Graphing live refractive IA readings and determining refractive stability translates to more accurate IOL selection.

## Introduction

Visual outcomes following cataract surgery are dependent on accurate biometry and intraocular lens (IOL) prediction formulae which account for effective lens position and the total astigmatism of the cornea. The latter is achieved by estimating/measuring the anterior and posterior corneal power/astigmatism. In recent years, three methods have emerged to determine posterior corneal astigmatism; empirical estimation of posterior corneal astigmatism using anterior corneal data, directly measuring posterior corneal astigmatism, and measuring aphakic total astigmatism intra-operatively.^1^^,^^2^ The latter involves the Optiwave Refractive Analysis System (ORA; Alcon, Fort Worth, TX, USA) which uses intra-operative aberrometry (IA) to determine the aphakic refraction via Talbot moiré interferometry.^2^^,^^3^ An optical wavefront passes through a pair of gratings and the diffraction producing a fringe pattern is analyzed to provide information on sphere, cylinder, and axis.^4^ Theoretically, because the cornea is the only refractive element in the aphakic state, this calculation accounts for surgically induced astigmatism and the impact of the posterior corneal astigmatism.^5^ As such, an astigmatism correcting toric IOL ranging in cylinder power (e.g. T3 corresponds to 1.5 diopters [D] in the IOL plane and 1.03 D in the corneal plane whereas T4 corresponds to 2.25 D IOL and 1.55 D in the corneal plane) can be implanted to decrease postoperative astigmatism.^6^

During an ORA “capture,” the surgeon obtains 40 readings and the average is used to calculate IOL power and astigmatism. The success of IA has varied in the published literature. A number of factors may influence measurements, such as the intraocular pressure (IOP) during intra-operative measurement, clarity of optical pathway, globe compression by the speculum, and lid position.^2^^,^^4^^,^^7^^,^^8^ The ocular surface during surgery can also differ from the pre-operative tear film and inconsistencies in intra-operative ocular hydration (IOH) may affect aphakic refraction.^9^ Too much or too little hydration could affect the continuous live refraction reading, which is not saved and affect the captured 40 readings. If the capture of 40 measurements is ill timed, then an inaccurate reading could be measured without the surgeon's knowledge and result in incorrect IOL recommendations.

Assuming other factors are controlled, measurement variability of intra-operative aphakic live refraction can act as an indicator of IOH and surface stability. No studies to date have examined this relationship between intra-operative refractive stability and postoperative refractive outcome. This study examines the stability of intra-operative live refraction and its impact on accurate IOL selection and quantify the minimum stable refraction needed to achieve best postoperative refraction.

## Methods

This study was a nonrandomized, consecutive retrospective chart review from a single center; one surgeon performed all the cases. The study was conducted in accordance with the National Statement on Ethical Conduct in Human Research (2007), the CPMP/ICH Note of Guidance on Good Clinical Practice and followed the tenets of the Declaration of Helsinki. Inclusion criteria included patients with uncomplicated cataract surgery and normal macula function. Exclusion criteria included previous refractive surgery, inflammations of the eye (e.g. uveitis), keratoplasty, corneal scars, history of dry eyes, and zonular weakness. Assessment of characteristics of patients/eyes was based on recorded pre-operative assessment including routine biometry, history, and workup.

**Figure 1.**
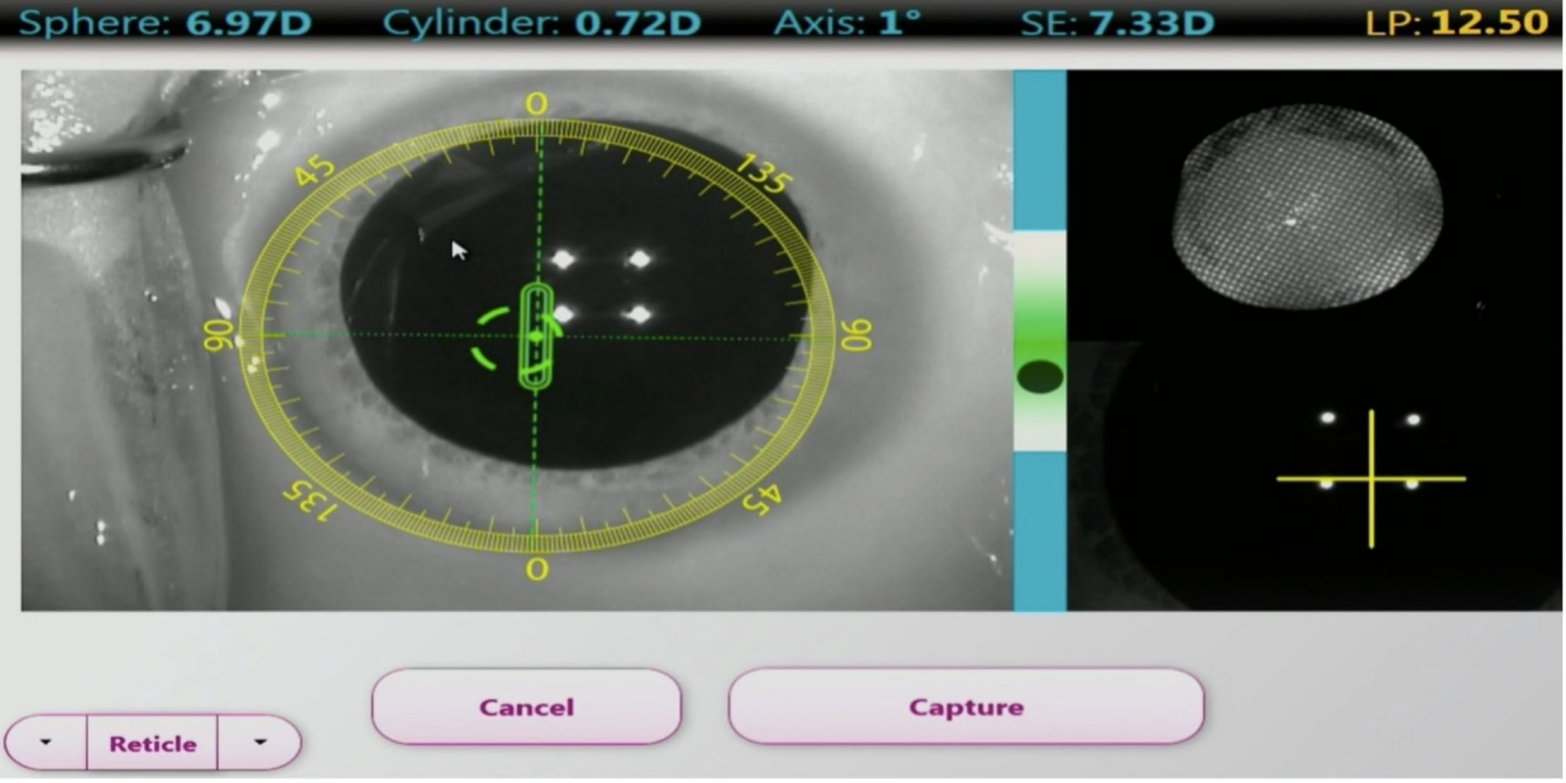
Intra-operative live aphakic refraction - reading shown at the *top* of the image. SE, spherical equivalent; LP, lens power.

**Figure 2.**
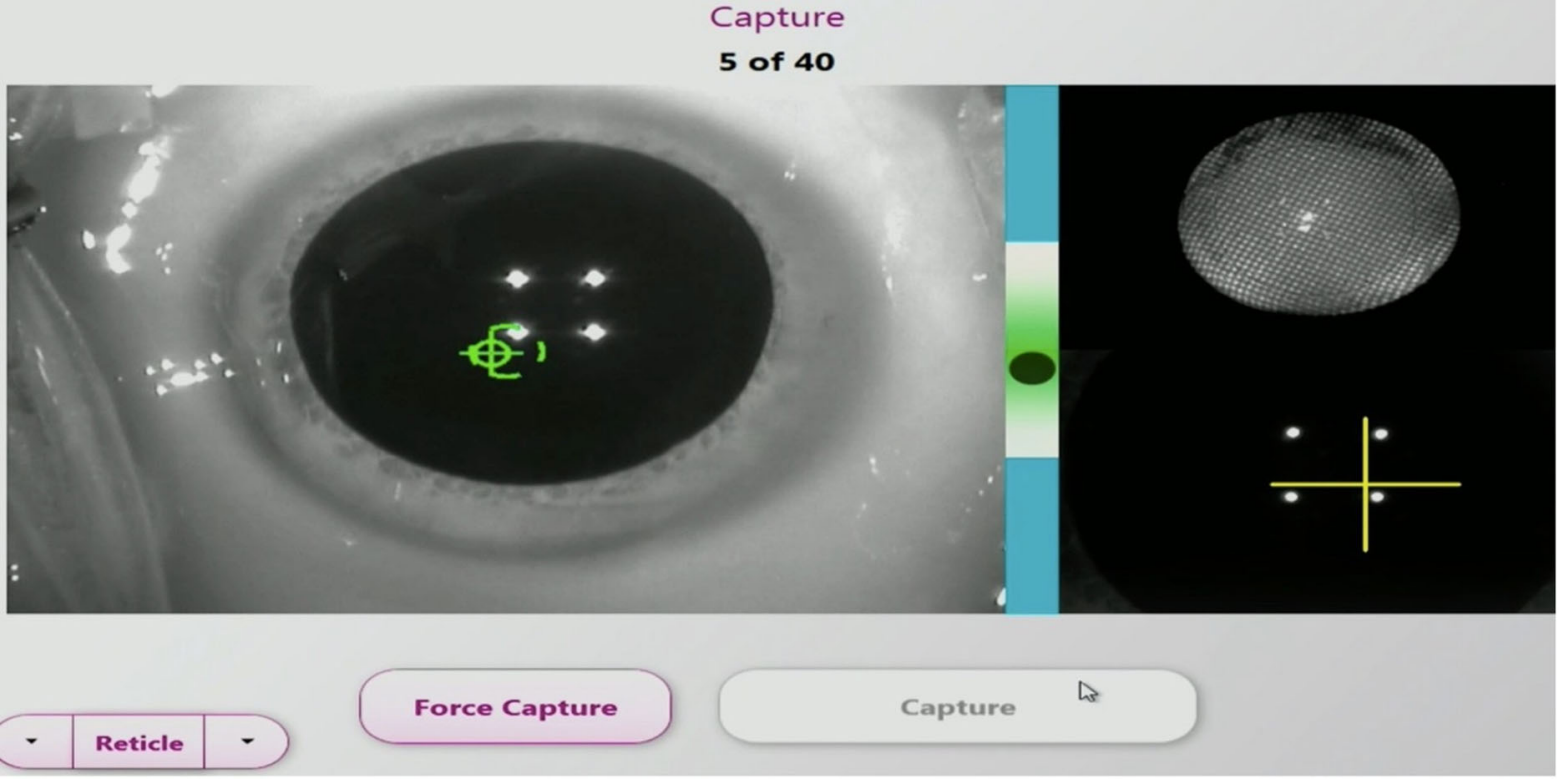
IA capture phase - 40 measurements are averaged but no live refraction is visible to the surgeon.

**Figure 3.**
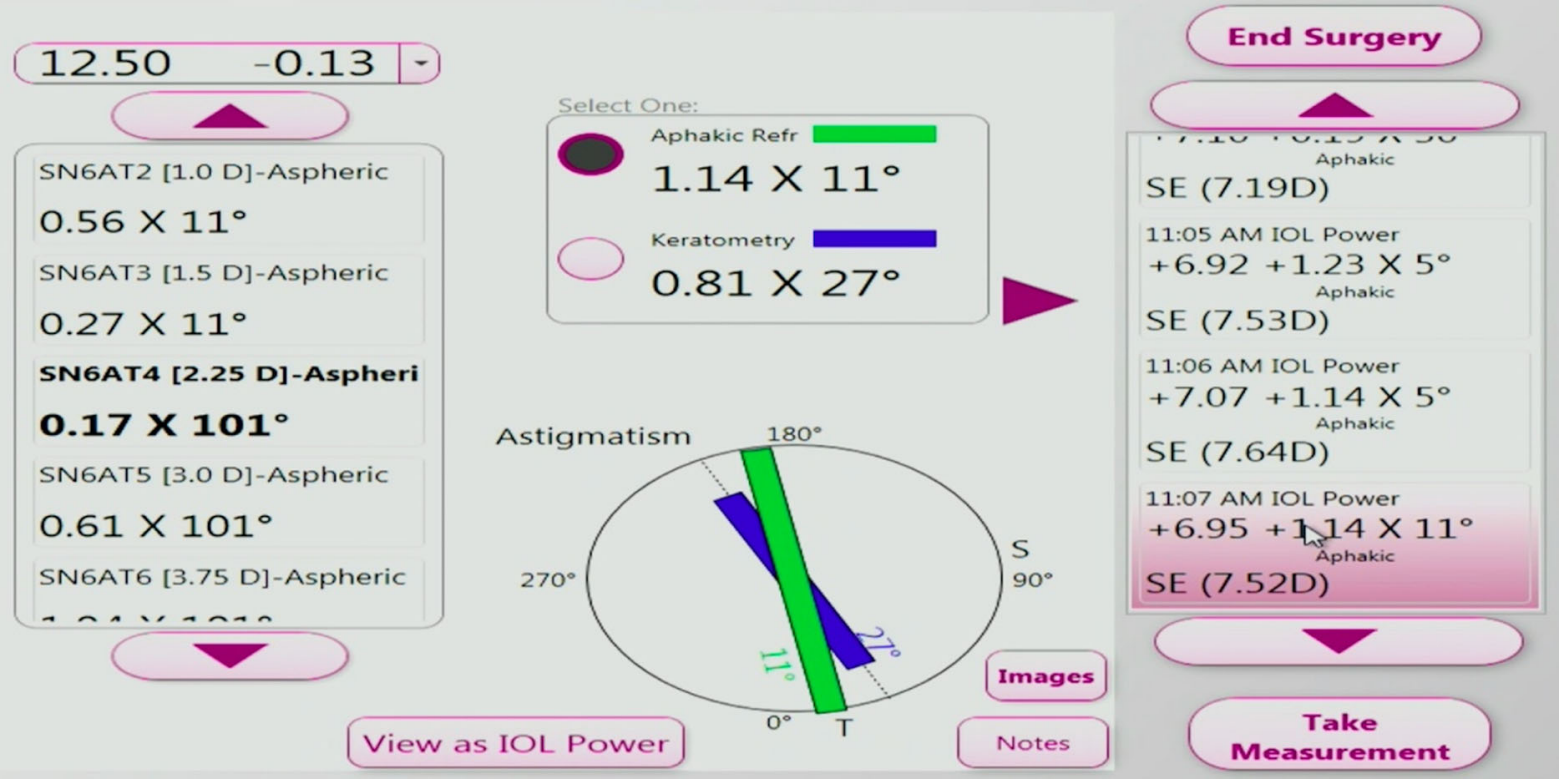
IA measurement based on IOL recommendation.

Video recordings of patients who had cataract surgery using ORA were reviewed. The ORA device continuously measures the refraction in the aphakic state prior to initiating the “capture” (Fig. 1) and insertion of the IOL. In the “capture” phase, the surgeon does not see the refraction values. Figure 2 demonstrates the capture process where 40 readings are taken and the recommended IOL power is then calculated (Fig. 3). The live refraction readings, unlike the capture data, are displayed but not stored. In Figure 1, the live refraction data suggests a cylinder of 0.72 D @1 degree whereas the capture data, which is an average of 40 measurements, suggests 1.14 D @11 degrees in Figure 3. In the present study, the live data were digitized from the video using optical character recognition software at a frame rate of 2.2 ms. Fifteen eyes of consecutive patients were selected for this study. Analysis was performed on 3 runs per eye and 18,000 data points were digitized, graphed, and analyzed. The predicted refractive error was compared with the 12-week postoperative refraction.

Operative variables that could affect the aberrometry were controlled by the surgeon according to standard clinical practice. The speculum was lifted away from the globe during measurement to prevent compression of the globe. Hydration of the cornea was maintained with balanced salt solution applied over the cornea and the IA measurement taken shortly after. A Barraquer tonometer was used to ensure the intraocular pressure was above 25 mm Hg across all patients, as recommended by the manufacturer. To minimize the effect of the tonometer on ocular hydration with contact, the cornea was hydrated across all patients with five drops of balanced salt solution (BSS). To ensure optical pathway clarity, care was taken to avoid bubbles within the viscoelastic in the central 4.5 mm and ProVisc (Alcon) was utilized. The anterior chamber was filled with cohesive viscoelastic in all patients, and there was no mixing of BSS or dispersive viscoelastic. No abnormalities such as vitreous floaters that may affect measurements were detected in our patient population.

### Stability of “Live” Aphakic Refraction

The live refraction was analyzed to estimate refractive stability and accuracy. An automated moving average method and a subjective methodology described below were used to determine the best outcome.

### Objective Automated Moving Average Method

Moving averages of cylinder values were calculated for different intervals from 10 frames (22 ms) to 100 frames (220 ms) for all 45 runs to determine the best minimum time frame of stable refraction that matched the predicted refraction. If the standard deviation was more than 0.5 D, that frame was excluded as unstable. For each interval of frames, the average cylinder value and amount of accepted data were calculated. The difference vector (DV) between the actual and the predicted postoperative refractive astigmatism for each of the different frame intervals was determined using the American Society of Cataract and Refractive Surgery astigmatism double angle plot tool.^10^ Significance was determined with an unpaired *t*-test.

**Figure 4.**
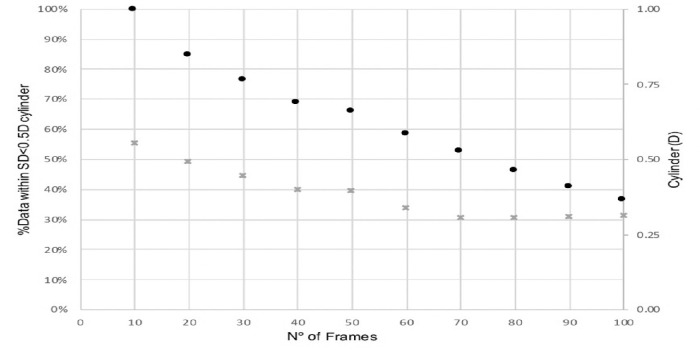
Two traces where moving average was used to calculate the average cylinder. *Black dots/grey crosses* represent two individual traces and the relationship between percentage of data with less than 0.5 D standard deviation at various frame lengths and the corresponding average cylinder.

Figure 4 shows the moving average data for a single run. If smaller frame intervals (e,g. 10 to 20 frames) are analyzed, a greater proportion of data had a standard deviation of <0.5 D, although the average cylinder was higher (i.e. data were noisier). In comparison for larger frame intervals (e.g. 90 to 100 frames), more data was excluded due to standard deviations >0.5 D and the average cylinder was lower.

### Subjective Methodology

**Figure 5.**
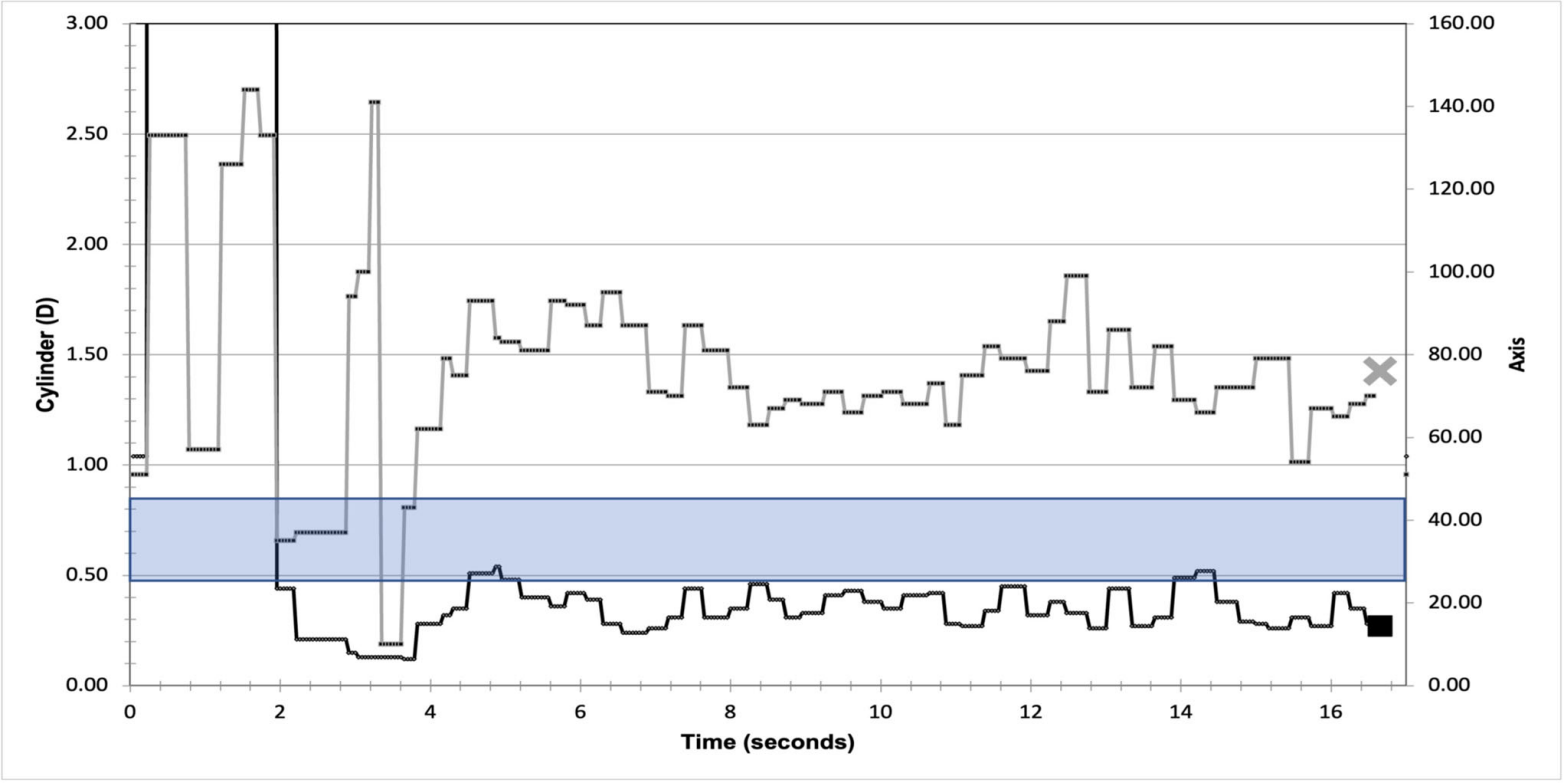
An example of a stable live refraction trace. The *grey line* represents the axis of measured astigmatism. The *grey cross* represents the value after the 40 captures. The *black line* represents cylinder measurement. The *black square* represents the average of the 40 captures. The band represents refraction which would result in a T2 IOL recommendation.

The digitized live refraction readings from each of the 45 IA videos were graphed (e.g. Fig. 5) to visually assess live refraction stability. These live tracings used “raw” data that was not grouped into frames as part of the objective automated moving average method. If a capture was completed during a period where readings remained between a range of 0.4 D cylinder for a time period of at least 5 seconds, it was classed as stable. The large peaks corresponding to BSS applications were excluded from the assessment. Utilizing these parameters, the runs were stratified into stable and unstable groups and a paired *t-*test was utilized to determine significance of DVs between these two groups for cylinder and spherical equivalent (SE). All analyses were performed using Microsoft Excel (version 16.37; Microsoft, Redmond, WA, USA) and the significance level was set at *P* = 0.05. Those with postoperative refraction <0.5 D cylinder were classed as expected, whereas those with an outcome of 0.5 D or greater cylinder were classed as unexpected.

### Power Analysis

This pilot study included 45 independent runs and 18,000 data points from 15 eyes of 15 participants with the goal providing valuable preliminary information while testing the characteristics and performance of this study design from a single center. Previous literature has recommended inclusion of at least 12 participants in pilot studies for planning clinical and translational research as this provides a balance between practicality and utility.^11^^–^^13^ In this study, 45 independent IA runs were used for statistical analysis. Whereas it is ideal to base sample size from a previous study, to the best of our knowledge, this is the first study to assess stability of IOH on IA reading stability. As such, sample size was determined based on preliminary results as a template using G-Power. To determine a true difference between the groups of 0.5, there needs to be 13 control subjects and 25 unstable groups to be able to reject the null hypothesis that the means are equal with a power of 0.5 and alpha of 0.05. As such, this study included 15 stable runs and 30 unstable runs, meeting the sample size criteria as a proof-of-concept study.

## Results

Forty-five individual runs were analyzed for 15 eyes in 15 patients. There were 7 men and 8 women with a mean age of 64.26 ± 12.95 years. The average postoperative cylinder was 0.2 D ± 0.47 D, average uncorrected visual acuity was −0.03 ± 0.1 logMAR, average corrected visual acuity was −0.09 ± 0.06 logMAR, and mean axial length was 24.38 ± 1.13 mm. In the 15 cases, 5 non toric, 2 T2, 3 T3, 3 T4, and 2 T8 IOLs were inserted. All surgeries were uncomplicated and no adverse events during or after the procedures were noted.

Empirically across all generated graphs between 10 frames (22 ms) to 100 frames (220 ms), 40 frames (88 ms), 50 frames (110 ms), and 60 frames (132 ms) had the best compromise between percentage of data, included and plateauing of cylinder averages and were applied to each run (see Fig. 4). The DVs for 40 frames (88 ms), 50 frames (110 ms), and 60 frames (132 ms) were compared with those obtained with the 40 “capture” intra-operative aberrometry reading. There was no significant difference between the DV of the stable portions of the live refraction and the captured reading (Table 1).

**Table 1.** Difference Vector in the Corneal Plane of IA Versus Frame Intervals

Group	DV (D)	*P* Value
IA versus measured post-operatively	0.43 ± 0.29	0.55
40 frames (88 ms) versus IA	0.51 ± 0.32	0.32
50 frames (110 ms) versus IA	0.47 ± 0.36	0.43
60 frames (132 ms) versus IA	0.49 ± 0.37	0.42

The subjective assessment of the live aphakic refraction showed that in 15 of 45 (33.3%) runs, the traces were qualitatively stable. In 30 of 45 runs (66.6%), the traces were assessed as unstable and the IOL recommendation should have been rejected and the capture repeated. Table 2 compares DVs of IA predicted cylinder during stable and unstable traces to actual postoperative measured cylinder. In stable runs, the DV was 0.27 D ± 0.15 D and was statistically significantly (*P* = 0.02) less than the DVs of the unstable group (0.47 D ± 0.28 D). The differences in SE for the stable and unstable groups were not statistically significant (*P* = 0.41).

**Table 2.** Difference Vectors for Cylinder and SE of Stable Versus Unstable IA Traces

Trace	*N* (Runs)	DV, Cylinder (D)	*P* Value (Cylinder)	DV, SE (D)	*P* Value (SE)
Stable	15	0.27 ± 0.15	0.02	0.03 ± 0.24	0.41
Unstable	30	0.49 ± 0.28		0.04 ± 0.28	

The 15 stable runs occurred across 7 eyes of 7 patients. In 14 of 15 (93.3%) of these stable traces, the predicted IOL power would have resulted in postoperative refractive outcomes <0.5 D cylinder and, in one case, 1 of 15 (6.7%), a 0.5 D cylinder outcome. Four aligned with pre-operative suggestions and in three cases, the toric power was altered by one unit (T2 ->T3, T3 ->T4, and T7 ->T8) from the pre-operative biometry suggestion. Figure 5 depicts an example of stable live refraction cylinder and axis trace from a single patient. The readings remained within 0.4 D range for at least 5 seconds and therefore the refraction was considered stable. The resultant measured cylinder was <0.5 D and a non toric IOL was implanted resulting in an unaided vision of 20/16. The DV between IA predicted and measured postoperative cylinder was 0.38 D.

**Figure 6.**
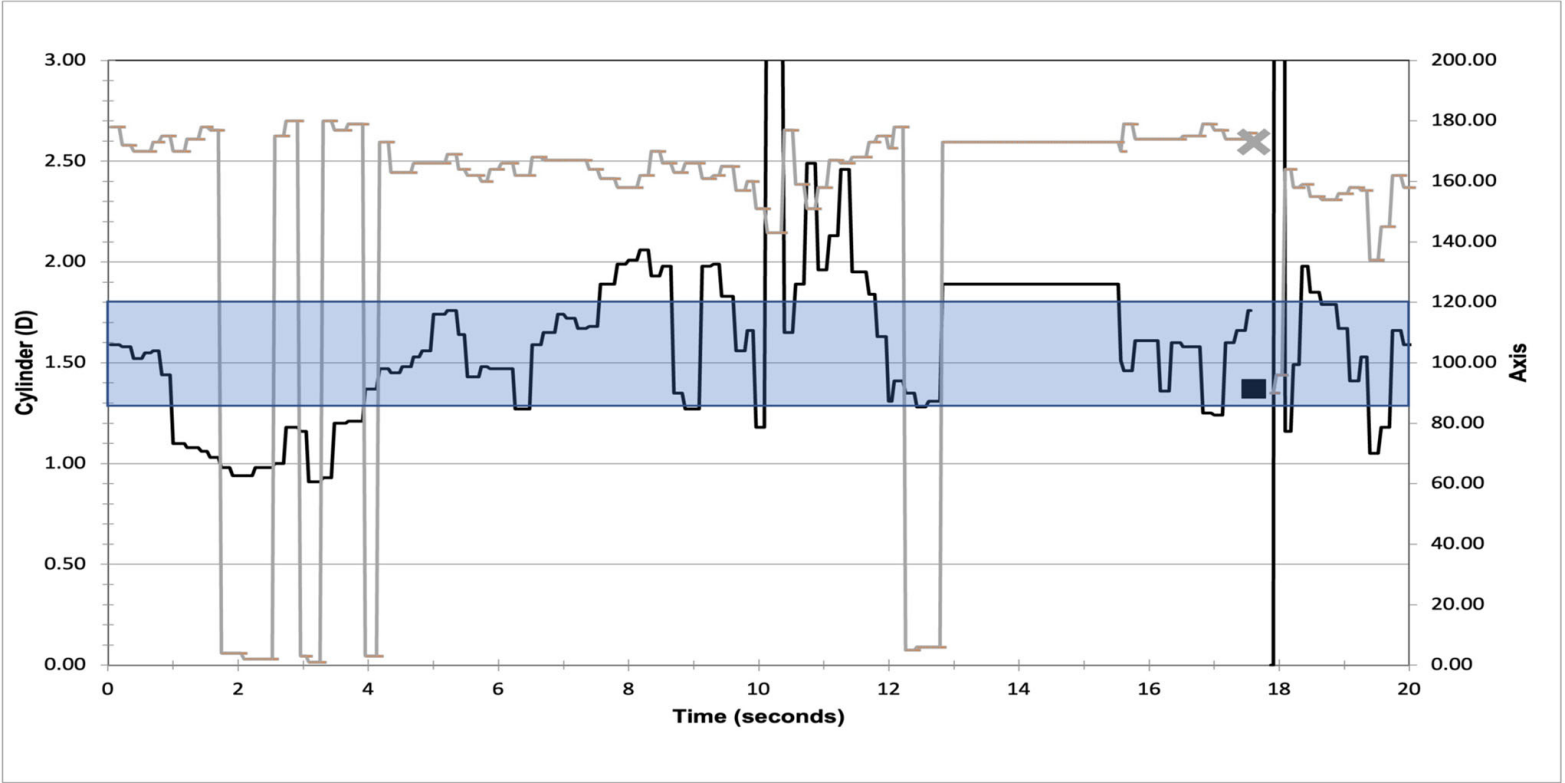
An example of an unstable live refraction trace. The *grey line* represents the axis of measured astigmatism. The *grey cross* represents the value after the 40 captures. The *black line* represents the cylinder measurement. The *black square* represents the average of the 40 captures. The band represents refraction which would result in a T4 IOL recommendation.

The subjectively classified unstable traces occurred in 30 of 45 runs in 11 eyes of 11 patients. In 14 of 30 (46.7%) traces, the predicted IOL power based on captured data still resulted in postoperative refractive outcomes <0.5 D cylinder, whereas in 16 of 30 (53.3%) an unexpected refraction of >0.5 D cylinder was seen. Although some patients had both stable and unstable runs, the potential impact was in 8 of 15 (53.3%) of cases without any stable runs. Three patients had IA predictions aligning with pre-operative suggestions (DV = 0.28 D ± 0.24 D), whereas in 5 patients, the toric power was altered (T2 -> T3, T3 -> T4, T7 -> T8, T2 -> T0, and T3 -> T0) with a DV of 0.60 D ± 0.29 D. Figure 6 demonstrates an IA trace of cylinder and axis values with large variations when the surgeon initiated a capture. As the trace was not within 0.4 for at least 5 seconds, this trace did not meet the criteria of a stable reading. In this case, the measured cylinder from the surgeon's capture was 1.37 D and a T4 toric IOL was implanted resulting in a DV between IA predicted and measured postoperative cylinder of 0.76 D and uncorrected visual acuity (VA) of 20/25.

Out of the 15 included eyes, 4 were long (>25 mm), 1 short (<22 mm), and the other 10 were normal axial length (22–25 mm). In long eyes, 9 of 12 runs (75%) compared to 2 of 3 (67%) of short eyes and 19 of 30 (63%) of normal eyes had unstable runs.

## Discussion

Measurement of the correct amount of astigmatism is important for accurate toric IOL choice. Intra-operative aberrometry during an aphakic state offers the opportunity to implant an IOL by incorporating the total corneal power (anterior and posterior) and the incision induced astigmatism.^2^ Whereas ocular surface hydration is a known variable effecting IA, it has not been examined in the past. This study attempted to optimize IA measurements using automation objectively and by subjectively observing the live refractive tracings.

Analysis found DVs for all frame intervals using automated methodology showed no significant difference when compared with IA readings. In 33.3% of cases, IA active refraction traces were stable and the DV (0.27 D ± 0.15 D) was significantly less than unstable traces (0.49 D ± 0.28 D). Alarmingly, 53.3% of unstable runs would have resulted in an unexpected refractive outcome >0.5 D of cylinder had the surgeon gone with the recommendation compared to only 6.7% of stable runs. Currently, this active refraction trace is not available and surgeons are consequently unaware of the potential impact of IOH has on the intra-operative measurement.

In this study, a process of statistical assessment of astigmatism stability was attempted by measuring the average value of a specific frame interval. If the standard deviation of the data was less than 0.5 D during that period, then the astigmatic value was included as a stable. Data were excluded as noise if the standard deviation was more than 0.5 D, as this could lead to a change in toric power. Different intervals were measured; 10 frames to 100 frames and the frames were moved along the trace, hence the moving average. In the ideal world, the perfect signal would have very little variability (i.e. very low standard deviation for the entire run). It would yield the same result and lens recommendation irrespective of frame size and location of the frame on a trace. Furthermore, all the data would be included in the analysis as the standard deviation would be below 0.5 D. However, the ocular surface does not sustain a stable refraction over long intervals with standard deviation below 0.5 D and significant portions of data are excluded as noise with long frames (see Fig. 4). Both drying of the cornea and perhaps frequent application of BSS likely contribute to these fluctuations. Figure 4 demonstrates that short intervals, alternatively, have many frames that achieve standard deviation of less than 0.5 D. This, however, fails to account for the general trend and includes more noisy data, leading to an overall higher cylinder prediction. A middle ground, where less data is lost and a tight standard deviation for a reasonable period can be achieved, would be optimal in an automated signal detection system.

Empirically across all generated graphs, 40 frames (88 ms), 50 frames (110 ms), and 60 frames (132 ms) had the best compromise. Figure 4 shows that around 40 to 60 frames there is a slight “plateau.” Beyond 60 frames there was a significant amount of data loss, and, at less than 40 frames, whereas a lot more data were included, it was likely to be noisier. Difference vectors to the actual postoperative cylinder from these frames’ interval average values were compared. Analysis showed there was no significant difference when comparing IA data provided by the device and the above frame interval analysis. Automation does not appear to help with signal detection in the ever-changing environment. Thereafter, we excluded the frame analysis method and utilized the data directly provided by the device to generate active refractive tracings.

For the first time, by digitizing live data, tracings of live refraction were created postoperatively. A trace was considered stable if the variability was less than 0.5 D, as seen with the naked eye. This threshold was set arbitrarily as 0.5 D or over could result in toric power change. Examining these IA refractive tracings quantitatively, 15 of 45 (33.3%) runs were stable and, in these cases, IA influenced IOL choices for better visual outcomes or confirmed the pre-operative selection (DV = 0.27 D). In the unstable runs, 14 of 30 (46.7%) cases would have resulted in a <0.5 D cylinder outcome, whereas in 16 of 30 (53.3%) of the cases, an unexpected refraction of >0.5 D cylinder would have resulted if the surgeon had gone with the recommendation. In none of these cases, the surgeon would have had access to the live refraction data at the time of IOL choice. Within the unstable runs, in the 5 cases when IA suggested a differing IOL power to the pre-operative choice, the potential resulting DV was 0.59 D, suggesting deferring to the pre-operative choice would yield better outcomes. In the other three cases, the IA suggestion was the same as pre-operative biometry. In these cases, the captures had occurred within the correct range by chance. IA is subject to ocular surface stability in an unblinking eye and its measurement can also vary and mislead the surgeon. The present study highlights the importance that the IOL choice is not an inactive choice from a printout but requires attention toward the quality of measurement irrespective of the source of the measurement.

The issue of toric IOL correction relies on the measurement of the anterior and posterior corneal astigmatism. IA offers the opportunity to measure a single value that includes anterior and posterior astigmatism and does not require a theoretical correction of posterior corneal astigmatism. This has clinical impact in the measurement of low against the rule astigmatism as theoretical formulae will suggest corrections. The low astigmatism value may be due to noise and thus correcting it would needlessly induce the cylinder leading to meridional magnification and spatial distortion should the surgeon act on it.^14^ For example, in low toric IOLs, such as T2, can induce astigmatism if non toric IOL is required. The variability in surface stability particularly in the unstable traces clearly highlights the importance of the capture occurring at the correct moment. In addition, presently there is no recommendation from manufacturers regarding the application of BSS to the cornea and the time period between application and measurement depends on the discretion of the surgeon. Incorporating the active refraction tracing into IA devices such that it can be reviewed live would better inform surgeons about the quality and stability of the readings. This is a simple yet useful solution to the current situation where the clinician is unaware of potential fluctuations.

By considering posterior astigmatism, IA can achieve a high rate of refractive outcome predictability especially in patients paying for presbyopic or toric IOLs as well as those with previous refractive surgery (e.g. patients with LASIK/post PRK).^15^^–^^19^ IA has also been shown to be valuable in the treatment of astigmatism during cataract surgery with both power selection and alignment of the toric IOLs and corneal astigmatic incision titration.^20^^–^^24^ However, in the literature, there have been varying results of IA accuracy with some studies advocating for caution when interpreting IA measurements with a significant discrepancy compared to pre-operative measurement.^25^^,^^26^ The range of prediction of postoperative cylinder <0.5 D of the target refraction ranges from 48.1% to 82% across multiple recent studies.^5^^,^^15^^,^^17^^,^^19^^,^^27^^–^^29^ Just as biometry may be affected by dry eyes, fixation, and size of palpebral aperture, a number of factors may confound intra-operative aberrometry measurements, such as IOP, distortion from eyelid speculum, fixation, and vitreous floaters.^2^^,^^4^^,^^7^^,^^30^ Studies have demonstrated that by being attentive, operative variables can be adequately controlled during aberrometry measurements and consistent across these studies is the researchers’ optimal efforts to keep surgical conditions constant to minimize confounding error from potential confounding variables (e.g. speculum pressure).^20^^,^^22^ However, there is no current way to measure ocular surface stability and, as such, without knowledge of the researcher, the surface may be too dry or overhydrated with BSS impacting the measurement. In cases such as these, confounding influences such as variation in IOH may contribute to aberrant readings without the surgeon's knowledge. This study showed 93% of stable runs would have resulted in postoperative refractive outcomes <0.5 D compared to 47% of unstable runs. It is likely that variability in the outcomes of multiple studies may be influenced by a spread of measurements taken at both stable and suboptimal states. Results could be greatly improved if the trace was made visible to the surgeon, reducing prediction error and translation to better outcomes for patients.

IA has been shown to be comparable and superior in instances to formulae in normal and short eyes while providing superior results in long eyes.^5^^,^^15^^,^^16^^,^^20^^,^^30^^,^^31^ With regard to stability of the ocular surface stratified by axial length, there was no bearing. In long eyes, 9 of 12 runs (75%) compared to 2 of 3 (67%) of short eyes and 19 of 30 (63%) of normal eyes had unstable runs. This shows that the axial length likely is unrelated to intraocular surface hydration which effected all lengths of eyes in similar proportions, emphasizing the importance of careful monitoring to obtain the best measurement. With regard to usability and the surgeon learning curve, one survey found 20% of 101 respondents reported it took more than 100 cases to feel comfortable with the ORA.^32^ However, 38% reported the transition in the first month, after completing fewer than 30 cases.^32^ Including an active trace would expedite this process and avoid incorrect selection of toric lens, which, in low toric cases, can instead induce astigmatism if a non toric was required.

This study has a number of limitations. Exclusion of patients with “abnormal eyes” was based on recorded pre-operative and intra-operative information, which may have omitted minor corneal irregularities or other variables that could have reduced the quality of ORA readings. In a prospective study, these variables could be captured using topography and eyes excluded. Additionally, a single surgeon from a single center performed all surgeries and a limited sample size of 15 patients were included. However, this was mitigated by including 45 individual IA runs and 18,000 data points for power analysis. This study is a pilot proof-of-concept study for future studies that should include greater sample sizes for increased power. Additionally, further research may choose to include the live refractive trace made available to the surgeon at the time of intra-operative aberrometry.

In summary, surgeons should take advantage of the live refraction they see on the screen and ensure it is stable before capturing data. Surgeons should look at the captured refraction and expect it to be similar to the live refraction in sphere, cylinder, and axis. If there is a significant difference, they should consider repeating the measurement.
